# Radiation protection in paediatrics age

**DOI:** 10.1186/1824-7288-40-S1-A62

**Published:** 2014-08-11

**Authors:** Sergio Salerno, Claudia Geraci

**Affiliations:** 1 Dipartimento di Biopatologia e Biotecnologie Mediche e Forensi, Università di Palermo, Italy

## 

Radiological protection in paediatric population from medical imaging is a subject promoted by various international associations and it is becoming a main field of interest. Paediatric patients have a significant risk from ionizing radiation (IR) following X-ray examination (chest, abdomen and skeletal segments), multidetector CT (MDCT) and PET imaging [1]. Their greater damage’s risk is due to: growing tissues with elevated turnover and high radio sensitivity cellular, the high water content which amplifies the damage, the small body size that involves the exposure of large areas associated with the expectation of long- life makes possible the development of diseases resulting from genetic damage [1]. Also there is a huge increase of exposure due to imaging is recorded in many country also in Italy, in emergency and is mainly performed in “non paediatric hospitals” often with adult setting of the machine [2]. The first type of radioprotection is the use of alternatives imaging techniques; ETG and MR offer, in expert hands, a vital potential diagnostic in complete safety radio protectionist [3]. The radiologist who elects to perform the X-ray examination must be certain that this is justified and run with minimal doses for the same diagnostic benefit (As Low As Reasonably Achievable: ALARA). In these patients the X-ray studies is characterized by repeated examinations and this results in high doses even if only one exam does not deliver high levels of IR. MDCT examinations when strictly necessary, should be optimized “patient fit” in terms of scanning parameters mAs and Kw (according to age and weight of the patient the paediatric patient range from 700gr to 90 kgs), well collimation and only the region of interest should be examined and multiple sequence should be avoided [4]. Is a common idea that using paediatric protocols and alternative imaging techniques are important for reduce dose [4] and many authors suggested that the Size-Specific Dose Estimates (SSDE) received by the patients should be included in the patient electronic medical record; paediatric radiologist and clinicians can use the SSDE when assessing risk versus benefit for the child prior to performance of a scan MDTC. The extensive use of ionizing radiation in paediatric age needs a profound balance between possible risks and clinical advantage by referring clinicians and a patient fit protocols adjusted to age and BMI of the patient by the radiologist.
